# Supplementary material for: Altered microRNA Transcriptome in Cultured Human Liver Cells upon Infection with Ebola Virus
Source: Int J Mol Sci. 2021 Apr 6;22(7):3792. doi: 10.3390/ijms22073792 (PMC8038836; doi:10.3390/ijms22073792)
Supplement: Supplementary file 1 [file ijms-22-03792-s001.zip › Supplementary_File/C_ GO_Analysis_Results/16-30nt_go_Reston-96h-Huh7_vs_Control-96h-Huh7_up.mature_mirna_targets/CC_FoldEnrichment.pdf]

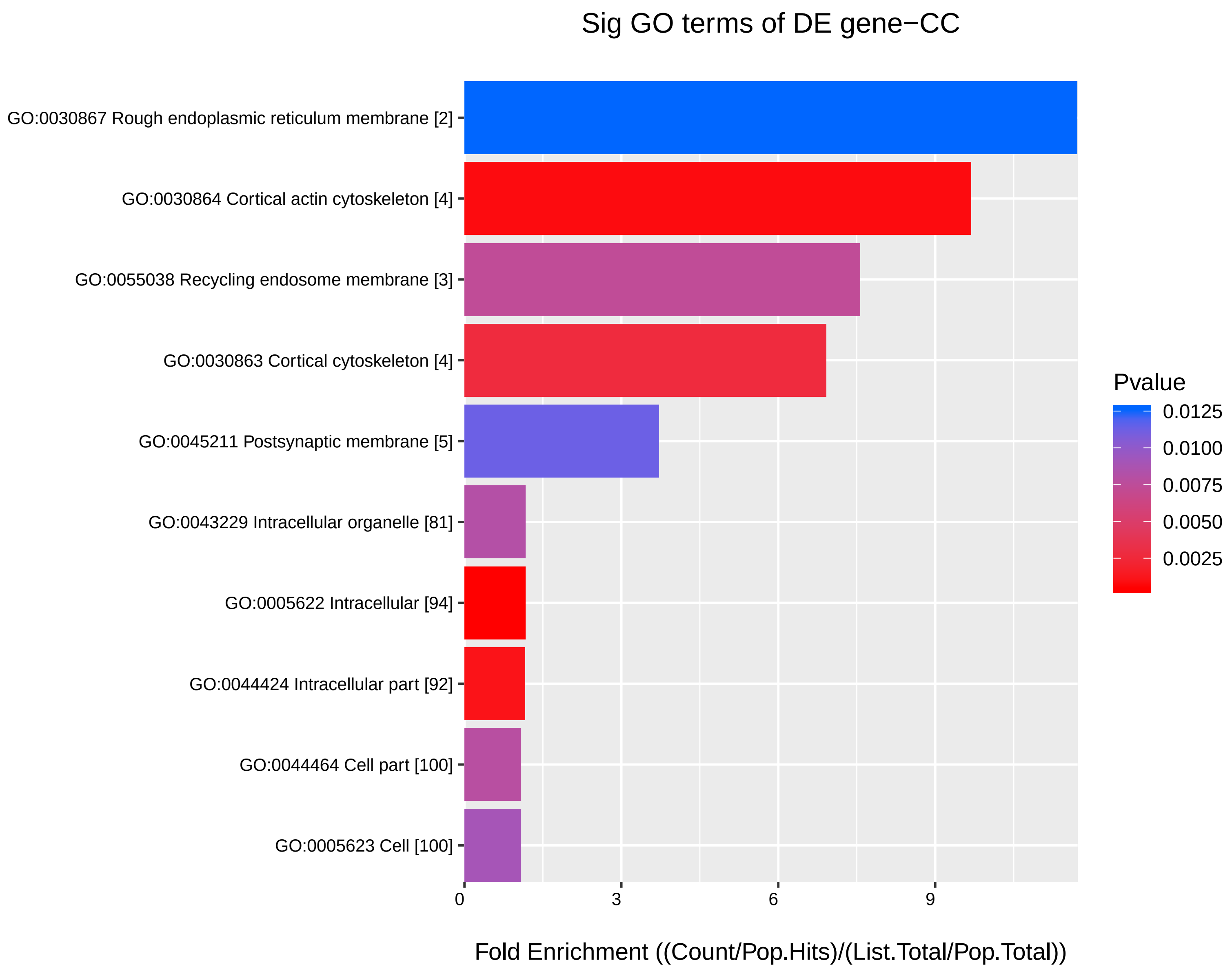

Sig GO terms of DE gene−CC
GO:0030867 Rough endoplasmic reticulum membrane [2]
GO:0030864 Cortical actin cytoskeleton [4]
GO:0055038 Recycling endosome membrane [3]
GO:0030863 Cortical cytoskeleton [4]
GO:0045211 Postsynaptic membrane [5]
GO:0043229 Intracellular organelle [81]
GO:0005622 Intracellular [94]
GO:0044424 Intracellular part [92]
GO:0044464 Cell part [100]
GO:0005623 Cell [100]
0
3
6
9
Fold Enrichment ((Count/Pop.Hits)/(List.Total/Pop.Total))
Pvalue
0.0125
0.0100
0.0075
0.0050
0.0025
